# Correlation between ocular residual astigmatism and anterior corneal astigmatism in children with low and moderate myopia

**DOI:** 10.1186/s12886-022-02560-2

**Published:** 2022-09-19

**Authors:** Jian Lin, Dexiang An, Yun Lu, Dongmei Yan

**Affiliations:** Lianyungang Maternal and Child Health Hospital, Lianyungang, 222000 Jiangsu China

**Keywords:** Ocular residual astigmatism, Anterior corneal astigmatism, Orthokeratology, Myopia

## Abstract

**Background:**

To assess the correlation between ocular residual astigmatism and anterior corneal astigmatism in children with low and moderate myopia.

**Methods:**

Refractive astigmatism was determined by subjective manifest refraction. Anterior corneal astigmatism was determined by IOL Master. Thibos vector analysis was used to calculate ocular residual astigmatism. Correlation analysis was used to assess the relationship between the amounts of ocular residual astigmatism and anterior corneal astigmatism. The relationship between the vectors of ocular residual astigmatism and anterior corneal astigmatism was evaluated by a physical method.

**Results:**

The study analysed 241 right eyes of 241 children aged 8 to 18 years old. In this study, the median magnitude of ocular residual astigmatism was 1.02 D, with an interquartile range was of 0.58 D. Against-the-rule ocular residual astigmatism was seen in 232 eyes (96.3%). There was a significant and moderate correlation between ocular residual astigmatism and anterior corneal astigmatism (*r* = 0.50, *P* < 0.001). Ocular residual astigmatism compensated for anterior corneal astigmatism in 240 eyes (99.6%). The mean compensation value was 1.00 ± 0.41 D (range 0.02 D to 2.34 D). Based on this effect, 37 eyes had a different axial classification of anterior corneal astigmatism and refractive astigmatism. In contrast, one eye (0.4%) had oblique ocular residual astigmatism and the ocular residual astigmatism superimposed with-the-rule anterior corneal astigmatism.

**Conclusions:**

The magnitude of ocular residual astigmatism was relatively large in myopic children and predominantly compensated for anterior corneal astigmatism. Ocular residual astigmatism should be assessed in patients before fitting them with orthokeratology lenses.

**Supplementary Information:**

The online version contains supplementary material available at 10.1186/s12886-022-02560-2.

## Background

Astigmatism is a common optical defect and prevails in human eyes [1–3]. It is defined as the difference in power between the steep and flat ocular meridians, which causes each point of an object to be refracted into two-line foci with specific orientations [2]. Significant astigmatism (≥ 1.0 D) reduces visual acuity; interferes with visual development and causes various symptoms such as glare, monocular diplopia, asthenopia, and distortion [2, 4]. Children with against-the-rule (ATR) astigmatism have a higher risk of myopia than those with with-the-rule (WTR) astigmatism [5]. It is more difficult to treat astigmatism compared to other refractive errors.

Refractive astigmatism (RA), anterior corneal (ACA), and ocular residual astigmatism (ORA) are different types of astigmatism. RA is the result of the combination of ACA and ORA. The ORA is composed of the posterior corneal surface, crystalline lens, a lesser extent vitreous, and retina with the perceptual physiology [1, 3, 6]. ORA is frequently calculated by the vectorial difference between RA and ACA. Previously studies have shown that ACA is mainly WTR astigmatism [3], whereas most of the ORA shows ATR astigmatism [3, 7]. Therefore, it is generally believed that the ORA provides compensatory effects for ACA [8–10]. However, to date, there are few detailed pieces of information on these effects.

Myopia is the most common ametropia, especially in school-aged children. Orthokeratology (Ortho-K) may be defined as reducing myopia temporarily in a planned way by the wearing of flat-fitting rigid contact lenses [11]. Modern overnight orthokeratology can diminish myopia rapidly, reliably, and reversibly [12]. Modern reverse-geometry orthokeratology lenses are mainly used to slow myopia progression and limit the extension of the ocular axis in children. It has been widely accepted as safe, effective, and reversible. The essence of orthokeratology is to shape the corneal epithelium. To be specific, orthokeratology causes central corneal epithelium thinning and mid-peripheral cornea epithelium thickening [13–15] without changing the posterior corneal radius [16, 17]. In other words, there is no change in posterior corneal astigmatism (PCA). While orthokeratology was not designed to correct astigmatism, it can change ACA. Multiple studies have demonstrated that orthokeratology can significantly reduce the ACA [18–20]. As a result, the ACA was reduced significantly after orthokeratology treatment. The ORA was unchanged and exposed. It can be speculated that the ORA was the main source of residual astigmatism after okeratoplasty. Orthokeratology did not correct RA reliably. Evidence suggests that residual astigmatism might be more problematic than expected if orthokeratology was used. So measuring ORA is equivalent to evaluating residual astigmatism that is not accounted for by the treatment. Studying the characteristics of ORA in myopic children can provide sound scientific evidence to support the conclusions. As mentioned previously, ORA is mainly ATR astigmatism, which has a higher risk of myopia than RA (mainly WTR astigmatism). On the other hand, knowledge of the distribution of the astigmatism components may be important to understand the development and progress of the eye’s refraction [2, 3]. The purpose of this study was to investigate the distributional characteristics of various types of astigmatism and assess the relationship between the vectors of ORA and ACA in children with orthokeratology indications. In addition, this study aimed to provide data for improving the effectiveness of orthokeratology treatment.

## Materials and methods

This study followed the tenets of the Declaration of Helsinki and was approved by the Lianyungang Maternal and Child Health Hospital review board. Informed consent was obtained from at least one parent of all participating children after an explanation of the nature of the study. For children aged 16 to 18, informed consent was also obtained from them directly.

### Participants selection

This cross-sectional study was conducted on 241 eyes of 241 subjects aged 8–18 years. Patients were included in this study if they had myopia from -5.00 to -1.00 D and regular astigmatism between -3.00 D to -0.25 D (ATR and oblique astigmatism are no less than -2.00 D), and best-corrected monocular visual acuity 20/20 or better. The exclusion criteria included any organic diseases of the eyes, such as cataract, glaucoma, keratoconus, irregular astigmatism, nystagmus, and children with strabismus. When RA was 0, it was unable to determine the properties of the RA axis. Therefore, they were also excluded. Finally, a total of 241 children met the inclusion criteria: 102 females and 139 males. The mean age was 11.8 ± 2.2 years. Only right eye data were included for analysis.

### Examination protocol and collection parameters

Standard subjective refraction tests were performed, and the RA was received by subjective manifest refraction. The ACA was the power difference between the steep and flat meridians on the anterior corneal surface. The IOL-Master 500 (Carl Zeiss, Meditec AG Jena, Germany) was used to measure anterior corneal curvature. Multiplying the curvature by 0.3375 to calculate corneal power. Three consistent measurements were collected and the averages were analysed [3].

### Data analysis and calculations

As described in our previous article [21], the positive cylinder notation is more consistent with the laws of physics and mathematics. Therefore, both RA and ACA are converted into the positive-cylinder notation before calculation. In addition, RA was transformed into the corneal plane before calculating ORA. Using Thibos vectored analysis to calculate the magnitude and axial direction of the ORA [22].

To describe the distribution of astigmatic axes, with-the-rule astigmatism was determined as positive-cylinder axes from 60° to 120°, and against-the-rule astigmatism was determined as positive-cylinder axes from 1° to 30° or 150° to 180°. Oblique astigmatism was defined as positive-cylinder axes from 31° to 59° or 121° to 149°.

### Analysis process of the relationship between the vectors of ORA and ACA

When the difference in the vector angle between the ORA and ACA was greater than 90° on the double angle vector diagram, the ORA compensated for the ACA [21]. The compensation values (CV) were calculated by multiplying ORA by cosine (180°- α), where α (range 90° to 180°) was the included angle between the vector of ORA and ACA on the double angle vector diagram.

### Statistical methods

SPSS statistics software package version 17.0 for Windows (IBM, Armonk, NY, USA) was used for the statistical analysis and calculations. Normality of all data samples was checked by means of the Kolmogorov–Smirnov test. The magnitudes of RA, ORA, spherical refraction and spherical equivalent refraction (SER) were non-normally distributed. The non-normality measurement data are expressed as the median value and interquartile range (IQR). The magnitude of ACA was normally distributed and was expressed as the mean ± standard deviation (SD). Correlation coefficients (Pearson or Spearman depending on whether normality conditions could be assumed) were used to assess the correlation between ORA and ACA, ORA and RA. Correlations were considered to be statistically significant when the associated *p*-value was < 0.05.

## Results

### Characteristics of the study population

Of the 241 patients (241 right eyes), 139 (57.7%) were male. The mean age of the patients was 11.8 years old (SD: 2.2; range: 8–18 years). The sphere refraction ranged from -5.00 D to -1.00 D (median value was -3.00 D, IQR was 2.0 D). The myopia was -3.00 or less in 54.8% (132 eyes). The SER ranged from -6.25 D to -1.13 D (median value was -3.38 D, IQR was 2.0 D). At the corneal plane, the ACA was 1.00 or more in 85.5% (206 eyes), with a mean ACA of 1.63 ± 0.62 (range 0.25 D to 3.54 D). The RA ranged from 0.22 D to 2.63 D (median value was 0.49 D, IQR was 0.46 D), 48 eyes (19.9%) had RA values of 1.00 D or more. The ORA ranged from 0.28 D to 2.48 D (median value was 1.02 D, IQR was 0.58 D), and it was 1.00 or more in 51.5% (124 eyes). Table 1 indicates the patient’s characteristics.

**Table 1:** Patient characteristics

Variable	Mean ± SD/Median(IQR)	Range
Age (y)	11.8 ± 2.2	8–18
Spherical refraction (D)	-3.00 (2.00)	-5.00—-1.00
RA (D)	0.49(0.46)	0.22–2.63
ACA (D)	1.63 ± 0.62	0.25–3.54
ORA (D)	1.02 (0.58)	0.28–2.48
SER (D)	-3.38(2.00)	-6.25—-1.13

*RA* Refractive astigmatism, *ACA* Anterior corneal astigmatism, *ORA* Ocular residual astigmatism, *SER* Spherical equivalent refraction

### Distribution of astigmatism

**Fig. 1:**
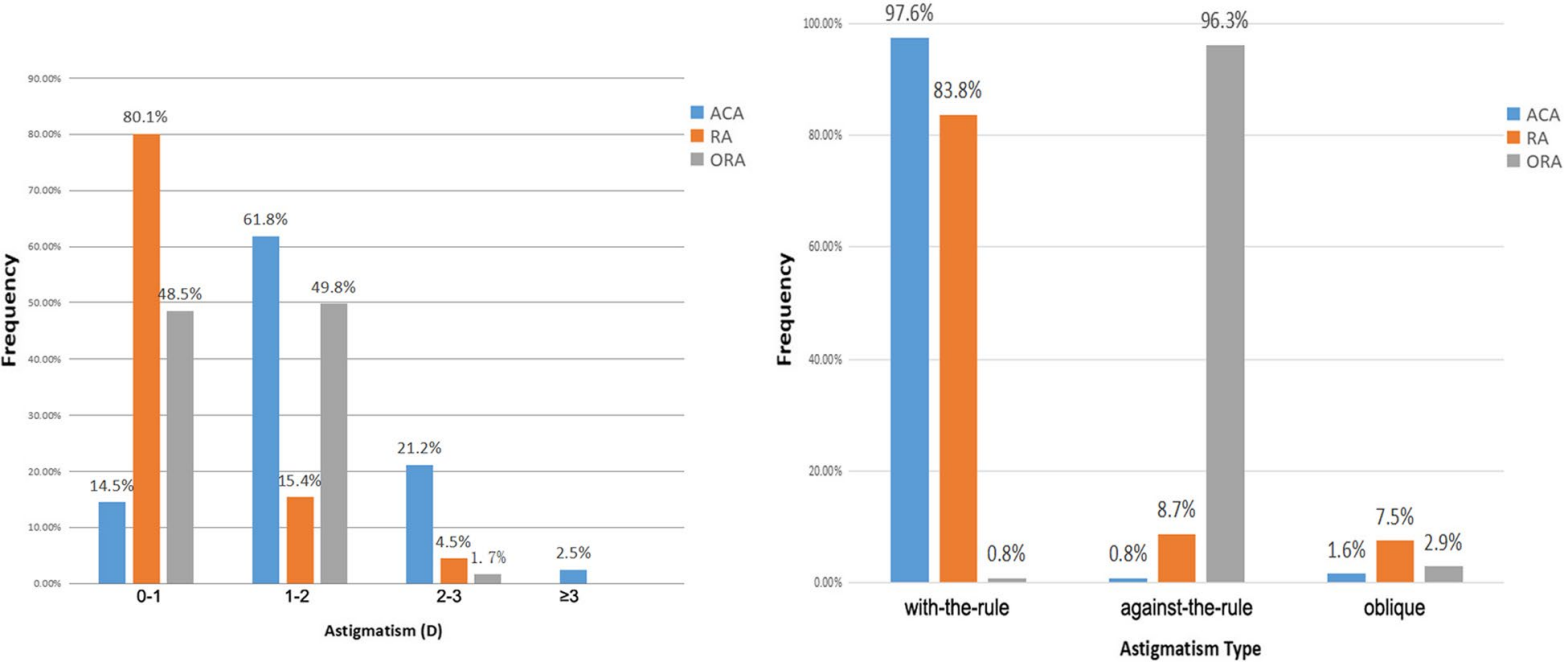
Distributions of different types of astigmatism

Figure 1 shows the distributions of astigmatism. The prevalence of RA (≥ 1.0 D) was 19.9%, ACA (≥ 1.0 D) 85.5%, and ORA (≥ 1.0 D) 51.5%. ACA had a peak prevalence (61.8%) between 1.0 D to 2.0 D (including 1D, excluding 2D). RA displayed a peak prevalence (80.1%) less than 1.0 D. ORA showed two peak prevalence rates (48.5% less than 1.0 D and 49.8% between 1.0 D to 2.0 D). Concerning axes, WTR ACA was observed in 235 eyes (97.6%), ATR ACA was seen in 2 eyes (0.8%), and oblique ACA was discovered in 4 eyes (1.6%). A total of 202 (83.8%) eyes showed WTR RA, 21 (8.7%) showed ATR RA, and 18 (7.5%) eyes showed oblique RA. A total of 232 eyes (96.3%) had ATR ORA, 2 eyes (0.8%) were WTR ORA, and 7 eyes (2.9%) showed oblique ORA.

### The correlation between ORA and ACA and between ORA and RA

**Fig. 2:**
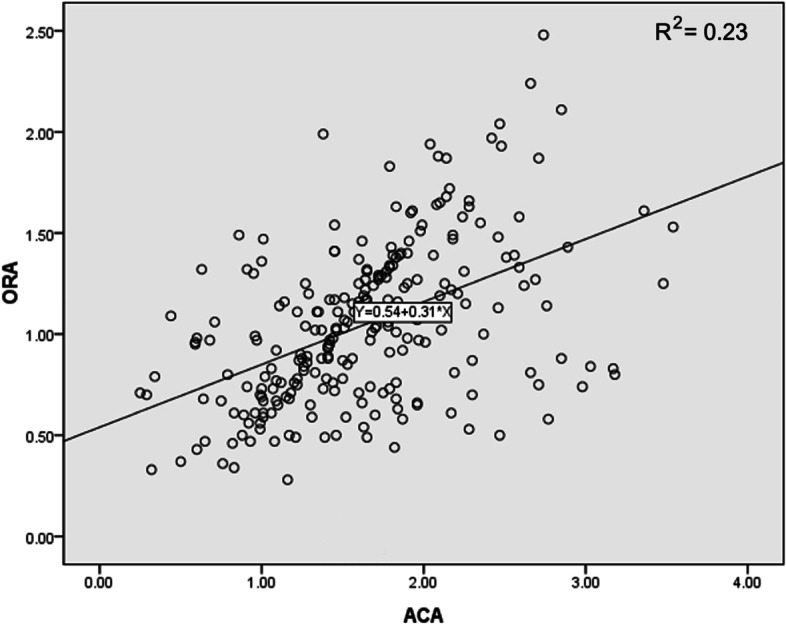
Correlation between the magnitude of ORA and ACA

There was no significant correlation between the magnitude of ORA and RA (*r* =—0.05, *P* = 0.42). There was a significant and moderate correlation between the magnitude of ORA and ACA (*r* = 0.50, *P* < 0.001). The predicting equations of the ORA from the magnitude of ACA were obtained (Fig. 2):

$$\mathrm{ORA}=0.54+0.31 \times \mathrm{ACA }\left({\mathrm{R}}^{2}=0.23\right)$$

### The relationship between the vectors of ORA and ACA

The ORA compensated ACA in 240 eyes (99.6%). Of these, 233 eyes (97.1%) showed ATR ORA, 5 eyes (2.1%) had oblique ORA, and 2 eyes (0.8%) were WTR ORA. The mean compensation value (CV) was 1.00 D (SD: 0.41 D, range 0.02 D to 2.34 D). The magnitude of CV/ACA was 0.25 or less in 14 eyes, 0.50 or less in 63 eyes, 0.75 or less in 179 eyes, and exceeded 1.00 in 16 eyes (Table 2). In contrast, only one eye showed a superimposed effect on ACA and had oblique ORA (ORA was 0.67 × 139). The superimposition value was 0.15 D, with ACA was 0.75 × 101. After the compensation effects of ORA to ACA, the axial classification of ACA and RA were different in 37 eyes. Specifically, a shift in axis from WTR ACA to ATR RA occurred in 17 eyes and from WTR ACA to oblique RA in 17 other eyes; also, there was a shift in axis from oblique ACA to WTR RA in 1 eye and from oblique ACA to ATR RA in 2 eyes. A total of 204 eyes had the same axial classification of ACA and RA. To be specific, 201 eyes had WTR ACA and WTR RA, 2 eyes had ATR ACA and ATR RA, and 1 eye showed oblique ACA and oblique RA.

**Table 2:** Distributions of the ratio of CV/ACA (*N*=240)

	The ratio of CV/ACA				
	≤ 0.25 (n)	≤ 0.50 (n)	≤ 0.75 (n)	≤ 1.00 (n)	> 1.00 (n)
WTR ACA (*n* = 234)	13	61	174	219	15
ATR ACA (*n* = 2)	0	0	2	2	0
Oblique ACA (*n* = 4)	1	2	3	3	1

*CV* Compensation values

### The relationship between the vectors of ORA and WTR ACA

In this study, WTR ACA was seen in 235 eyes. Of them, 230 eyes (97.9%) had ATR ORA, and 5 eyes (2.1%) had oblique ORA. The ORA in 234 eyes (99.6%) had compensatory effects on WTR ACA. Of these, 230 eyes (98.3%) showed ATR ORA, and 4 eyes (1.7%) were oblique ORA. The mean compensation value was 1.01 D (SD: 0.40 D, range 0.20 D to 2.34 D). The magnitude of CV/ACA was 0.25 or less in 13 eyes, 0.50 or less in 61 eyes, 0.75 or less in 174 eyes, and exceeded 1.00 in 15 eyes (Table 2). On the other hand, one eye with oblique ORA showed a superimposed effect on WTR ACA.

### The relationship between the vectors of ORA and ATR ACA, ORA and oblique ACA

One eye with oblique ORA and one eye with WTR ORA played compensatory effects on ATR ACA. Three eyes with ATR ORA and one eye with WTR ORA exhibited compensatory effects on the oblique ACA (Table 3).

**Table 3:** The effects of ORA on ACA (*N*=241)

	ORA					
	Compensatory effects			Superimposed effects		
	WTR	ATR	Oblique	WTR	ATR	Oblique
WTR ACA (*n* = 235)	0	230	4	0	0	1
ATR ACA (*n* = 2)	1	0	1	0	0	0
Oblique ACA (*n* = 4)	1	3	0	0	0	0

## Discussion

In the subjects with low and moderate myopia, the prevalence of RA (≥ 1.0 D) was 19.9%, ACA (≥ 1.0 D) 85.5%, and ORA (≥ 1.0 D) 51.5%. The mean ACA was 1.62 D (SD 0.62 D), the median (IQR) RA was 0.49 (0.46), and the median(IQR) ORA was 1.02 (0.58). Compared with the whole categories of refractive states (including emmetropia and ametropia), the prevalence of RA ≥ 1.0 D (19.9%) was similar to those reported in central China (17.4%) [3] and Singapore (19.2%) [22] but lower than those reported in Hong Kong (28.4%) [23] and Taiwan (32.6%) [24]. However, the prevalence of ACA (85.5%) and ORA (51.5%) were significantly higher than those studies [3, 25]. Meanwhile, the magnitude of ORA was larger than that in other studies. Li et al.[3] analysed 1783 12-year-old students and reported that the mean ORA was 0.72 D. Huynh et al. [8] found that the mean ORA was 0.76 D in 6-year-old children. One possible reason for this result is that the magnitude of ACA and ORA in myopic eyes was significantly larger than that in emmetropic and hyperopic eyes. Another possibility is that the compensation effects of ORA to ACA in myopia were more significant than those in other refractive states.

The prevailing wisdom was that the ORA compensated ACA [8–10]. Although our recent work evaluated the contribution of ORA to ACA in 5-year-old children [21], there is still no detailed information on the compensation effects in myope children. There was a significant and moderate correlation between the magnitude of ORA and ACA (*r* = 0.50, *P* < 0.001). The ORA had compensatory effects on the ACA in 240 eyes (99.6%).In 6.7% (16/240) of eyes, the compensation values exceeded the magnitude of the ACA. The axial classification of the ACA and RA were different after the compensation effects in 15.4% (37/240) of eyes. For 235 eyes with WTR ACA, 99.6% (234/235) of the ORA worked to offset it. Both ATR and oblique ORA can counteract WTR ACA, while oblique ORA can also superimpose it. The results were similar to our recent study in 5-year-old children with significant astigmatism [21]. With regard to ATR ACA, both WTR and oblique ORA had compensatory effects on it. WTR and ATR ORA can counteract oblique ACA.

Actually, the correlation between ORA and ACA and between ORA and RA would vary according to the population being studied (such as age, sphere, cylinder, etc.). We found a moderate and positive correlation between ORA and ACA (*r* = 0.50, *P* < 0.001) in low and moderate myopic eyes. Wallerstein and colleagues [7] obtained similar outcomes (*r* = 0.44) by studying 21,580 myopic eyes. Nevertheless, our previous study found a much weaker correlation between ORA and ACA (*r* = 0.17) by studying 14 emmetropic eyes, 68 myopic eyes, and 19 hyperopic eyes [21]. Moreover, there were no significant correlations between ORA and RA (*r* =—0.05, *P* = 0.42) in this study. Piñero et al. also reported a negative but nonsignificant correlation (*r* = -0.01, *P* = 0.89) by studying 14 emmetropic eyes, 68 myopic eyes, and 19 hyperopic eyes [6]. A positive correlation was found in other studies [1, 7]. Our previous study found a significant and negative correlation between ORA and RA (*r* = -0.27, *P* = 0.001) [21]. Characteristics of the studied population, such as age, sphere, cylinder, etc., may be the reason for the different research results.

Multiple studies have demonstrated that orthokeratology causes significant changes in corneal astigmatism [18–20]. Mountford and Pesudovs[18] stated that 87.0% of patients with reverse geometry orthokeratology lenses had some reduction of ACA in their study. Chan [19] reported reductions in ACA of up to -2.50 D three weeks after toric orthokeratology treatment. Chen et al.[20] investigated 35 myopic children with moderate-to-high astigmatism and found a 79% reduction in ACA after one month of toric orthokeratology. As mentioned previously, orthokeratology did not change the posterior corneal radius [16, 17]. Consequently, the ORA was unchanged and exposed after orthokeratology treatment. Relatively large amounts of ORA, which is mainly against-the-rule astigmatism, existed in 12-year-old children with orthokeratology indications, which may be one of the reasons for the degraded visual quality after orthokeratology. Sorbara et al. [26] found that the proportion of subjects with spectacles reaching 6/6 or better visual acuity was higher than those wearing orthokeratology lenses ( 89% vs. 83%). Unfortunately, there have been no studies on the relationship between ORA and orthokeratology. Future research needs to be done on the correlation between ORA and residual astigmatism after orthokeratology and on the specific influence of ORA on visual quality after orthokeratology.

In conclusion, for low- and moderate-myopia eyes, we found that the prevalence of ORA (≥ 1.0 D) was relatively high, and the magnitude was large. Nearly all (99.6%) ORA compensated for ACA, the magnitude of CV/ACA exceeded 1.00 in 6.7%(16/240) of eyes, and 15.4% (37/240) of eyes had a different axial classification of ACA and RA after the compensation effects. The ACA was reduced significantly, yet the ORA was unchanged and exposed after orthokeratology treatment. Therefore, ORA was the main source of residual astigmatism after okeratoplasty. Evidence suggests that residual astigmatism might be more problematic than expected if orthokeratology was used. Measuring ORA is equivalent to evaluating residual astigmatism that is not accounted for by the treatment. Therefore, the ORA should be assessed first before the completion of a course of orthokeratology. It may be a useful indicator to decide which patient is a good candidate for orthokeratology (those with low ORA). In addition, more attention should be given to the specific influence of ORA on the effectiveness of orthokeratology.

## Supplementary Information

**Additional file 1**.

## Data Availability

All data generated or analysed during this study are included in its supplementary information files.

## Abbreviations

ORA: Ocular residual astigmatism
ACA: Anterior corneal astigmatism
RA: Refractive astigmatism
WTR: With the rule
ATR: Against the rule
SER: Spherical equivalent refraction
IQR: Interquartile range
SD: Standard deviation
CV: Compensation values
